# KNb_1.75_V_0.25_PS_10_

**DOI:** 10.1107/S1600536811004430

**Published:** 2011-02-12

**Authors:** Jaemin Yu, Hoseop Yun

**Affiliations:** a Division of Energy Systems Research and Department of Chemistry, Ajou University, Suwon 443-749, Republic of Korea

## Abstract

The title compound, potassium diniobium vanadium phospho­rus deca­sulfide, KNb_1.75_V_0.25_PS_10_, was obtained by reaction of the elements with a eutectic mixture of KCl/LiCl. It is isostructural with the quaternary KNb_2_PS_10_, but the Nb sites are occupied by statistically disordered Nb (87.5%) and V (12.5%) atoms. The structure is composed of anionic _∞_^1^[*M*_2_PS_10_]^−^ chains (*M* = Nb/V) separated from each other by K^+^ ions. The chain is composed of [*M*S_8_] distorted bicapped trigonal prisms and [PS_4_] tetra­hedra. There are no inter­chain bonding inter­actions. The crystal used for the X-ray analysis was a racemic twin.

## Related literature

For related ternary compounds, see: Brec *et al.* (1983*a* ▶,*b* ▶). For related quaternary compounds, see: Goh *et al.* (2002 ▶); Do & Yun (1996 ▶); Kim & Yun (2002 ▶); Kwak *et al.* (2007 ▶); Bang *et al.* (2008 ▶); Do & Yun (2009 ▶). For related penta­nary compounds, see: Kwak & Yun (2008 ▶); Dong *et al.* (2005*a* ▶,*b* ▶); Park & Yun (2010 ▶). For typical Nb^4+^—Nb^4+^ bond lengths, see: Angenault *et al.* (2000 ▶)

## Experimental

### 

#### Crystal data

KNb_1.75_V_0.25_PS_10_
*M* *_r_* = 2264.39
Orthorhombic, 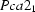

*a* = 12.9696 (3) Å
*b* = 7.5229 (2) Å
*c* = 13.3248 (4) Å
*V* = 1300.09 (6) Å^3^
*Z* = 1
Mo *K*α radiation
μ = 3.73 mm^−1^
*T* = 290 K
0.36 × 0.06 × 0.06 mm

#### Data collection

Rigaku R-AXIS RAPID diffractometer
Absorption correction: multi-scan (*ABSCOR*; Higashi, 1995 ▶) *T*_min_ = 0.649, *T*_max_ = 1.000
11855 measured reflections
2970 independent reflections
2859 reflections with *I* > 2σ(*I*)
*R*_int_ = 0.028

#### Refinement

*R*[*F* ^2^ > 2σ(*F* ^2^)] = 0.019
*wR*(*F* ^2^) = 0.042
*S* = 1.07
2970 reflections
130 parameters
1 restraint
Δρ_max_ = 0.49 e Å^−3^
Δρ_min_ = −0.28 e Å^−3^
Absolute structure: Flack (1983 ▶), 1417 Friedel pairs
Flack parameter: 0.47 (4)

### 

Data collection: *RAPID-AUTO* (Rigaku, 2006 ▶); cell refinement: *RAPID-AUTO*; data reduction: *RAPID-AUTO*; program(s) used to solve structure: *SHELXS97* (Sheldrick, 2008 ▶); program(s) used to refine structure: *SHELXL97* (Sheldrick, 2008 ▶); molecular graphics: locally modified version of *ORTEP* (Johnson, 1965 ▶); software used to prepare material for publication: *STRUCTURE* *TIDY* (Gelato & Parthé, 1987 ▶) and *WinGX* (Farrugia, 1999 ▶).

## Supplementary Material

Crystal structure: contains datablocks global, I. DOI: 10.1107/S1600536811004430/fj2392sup1.cif

Structure factors: contains datablocks I. DOI: 10.1107/S1600536811004430/fj2392Isup2.hkl

Additional supplementary materials:  crystallographic information; 3D view; checkCIF report

## Figures and Tables

**Fig. 1.**
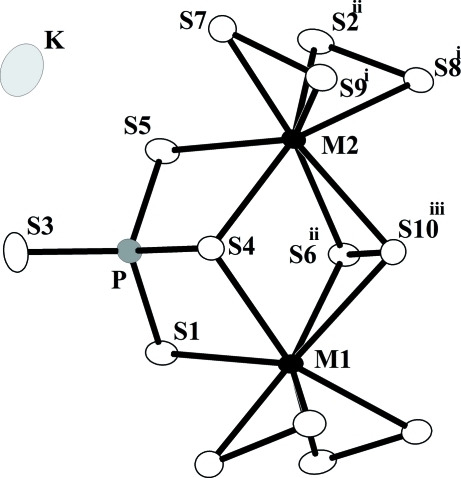
A view of the bicapped trigonal biprismatic [M2S12] unit (M=V/Nb) and its neighboring tetrahedral [PS4] groups. Open circles are S atoms, filled circle are Nb atoms, dark and pale gray circles are P and K atoms, respectively. Displacement ellipsoids are drawn at the 50% probability level. [Symmetry code: (i) 1 - x, -y, -1/2 + z; (ii) 0.5 - x, -y, -1/2 + z; (ii) -1/2 + x, -y, z]

**Fig. 2.**
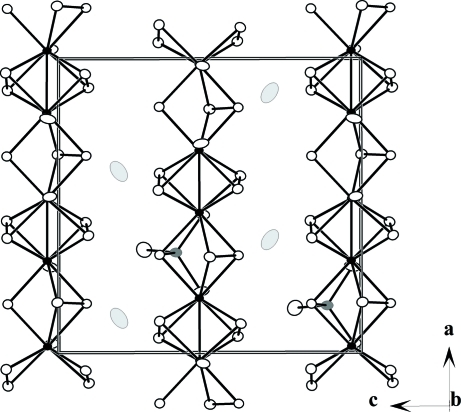
View of the KNb2 -xVxPS10 down the b axis showing the one-dimensional nature of the compound. Atoms are as marked in Fig. 1.

## supplementary crystallographic information

### Comment

Ternary group 5 metal thiophosphates have been reported to have mostly low-dimensional structures. Especially the Nb_2_PS_10_ phase has a two-dimensional layered structure (Brec *et al.*, 1983*a*) and V_2_PS_10_ adopts a one-dimensional chain structure (Brec *et al.*, 1983*b*). Due to empty spaces and the orbitals which can accommodate electrons, they have been of potential importance as cathode materials for secondary batteries and a number of quaternary alkali metal Nb thiophosphates, ANb_2_PS_10_ (A=monovalent metals) have been investigated. Among them are NaNb_2_PS_10_ (Goh *et al.*, 2002), KNb_2_PS_10_ (Do & Yun, 1996), RbNb_2_PS_10_ (Kim & Yun, 2002), CsNb_2_PS_10_ (Kwak *et al.*, 2007), TlNb_2_PS_10_ (Bang *et al.*, 2008), Ag_0.88_Nb_2_PS_10_ (Do & Yun, 2009), K_0.34_Cu_0.5_Nb_2_PS_10_ (Kwak & Yun, 2008), K_0.5_Ag_0.5_Nb_2_PS_10_ (Dong *et al.*, 2005*a*), Rb_0.38_Ag_0.5_Nb_2_PS_10_ (Dong *et al.*, 2005*b*), and Cs_0.5_Ag_0.5_Nb_2_PS_10_ (Park & Yun, 2010). It is interesting that no V analogue of these phases has been discovered yet. As a result of efforts to find new phases in this family, we report the synthesis and characterization of a new mixed-metal quintenary thiophosphate, KNb_2 -_*_x_*V*_x_*PS_10_ (*x*=0.25).

The structure of KNb_2 -_*_x_*V*_x_*PS_10_ is isostructural with the quaternary KNb_2_PS_10_ and detailed description of the structure is given previously (Do & Yun, 1996). The title compound is made up of the bicapped trigonal biprismatic [*M*_2_S_12_] unit (*M*=Nb/V) and the tetrahedral [PS_4_] group. The *M* sites are occupied by the statistically disordered Nb(~87.5%) and V(~12.5%) atoms. The bicapped biprismatic [*M*_2_S_12_] units and its neighboring tetrahedral [PS_4_] groups are given in Figure 1. These [*M*_2_S_12_] units are linked together to form the one-dimensional chains by sharing the S_2_^2-^ prism edge. The one-dimensional chain composed of *M*, P, and S extends along [100] and can be described as ∞^1^[*M*_2_PS_10_^-1^].

The *M* atoms associate in pairs with M—*M* interactions alternating in the sequence of one short (2.8851 (3) Å) and one long (3.7590 (3) Å) distances. The short distance is typical of Nb^4+^—Nb^4+^ bonding interactions (Angenault *et al.*, 2000). There are no interchain bonding interactions except the van der Waals forces and the K^+^ ions in this van der Waals gap stabilize the structure through the electrostatic interactions (Figure 2). Finally, the classical charge balance of this phase can be represented by [K^+^][*M*^4+^]_2_[PS_4_^3-^][S_2_^2-^]_3_ and this is consistent with the highly resistive and diamagnetic nature of the compound.

### Experimental

The compound KNb_2 -_*_x_*V*_x_*PS_10_ was prepared by the reaction of the elemental Nb, V, P, and S with the use of the reactive alkali metal halides. A combination of the pure elements, Nb powder (CERAC 99.9%), V powder (CERAC 99.5%), P powder(CERAC99.95%), and S powder (Aldrich 99.999%) were mixed in a fused silica tube in a molar ratio of Nb: V: P: S = 1:1:1:5 with the eutectic mixture of KCl/LiCl. The mass ratio of the reactants and the halides flux was 2:1. The tube was evacuated to 0.133 Pa, sealed and heated gradually (50 K/h) to 650 K, where it was kept for 72 h. The tube was cooled to 423 K at 3 K/h and then was quenched to room temperature. The excess halides were removed with distilled water and black needle shaped crystals were obtained. The crystals are stable in air and water. A microprobe analysis of the crystals was made with an EDAX equipped scanning electron microscope (Jeol JSM-6700 F). Analysis of these crystals showed only the presence of K, Nb, V, P, and S. A quantitative analysis performed with standards gave the ratio of Nb: V = 87: 13, which corresponds to KNb_1.74_V_0.26_PS_10_.

### Refinement

The refinement of the model with occupational disorder on the *M* site caused significant decrease of the *R*-factor (wR2 = 0.042) in comparison if the full occupation by either metal had been considered (wR2 > 0.05). Also the displacement parameters in the disordered model became plausible. The disordered atoms were supposed to have the same displacement parameters. The nonstoichiometry of the K site was checked by refining the occupancy of K while those of the other atoms were fixed. With the nonstoichiometric model, the parameter remained the same. The large anisotropic displacement parameters for alkali metals are also found in the related compounds such as KNb_2_PS_10_ (Do & Yun, 1996). The highest residual electron density is 0.86 Å from the M2 site and the deepest hole is 0.85 Å from the M1 site.

### Crystal data

**Table:**

KNb_1.75_V_0.25_PS_10_	*F*(000) = 1086
*M**_r_* = 2264.39	*D*_x_ = 2.892 Mg m^−^^3^
Orthorhombic, *P**c**a*2_1_	Mo *K*α radiation, λ = 0.71073 Å
Hall symbol: P 2c -2ac	Cell parameters from 11171 reflections
*a* = 12.9696 (3) Å	θ = 3.1–27.5°
*b* = 7.5229 (2) Å	µ = 3.73 mm^−^^1^
*c* = 13.3248 (4) Å	*T* = 290 K
*V* = 1300.09 (6) Å^3^	Needle, black
*Z* = 1	0.36 × 0.06 × 0.06 mm

### Data collection

**Table:**

Rigaku R-AXIS RAPID diffractometer	2859 reflections with *I* > 2σ(*I*)
ω scans	*R*_int_ = 0.028
Absorption correction: multi-scan (*ABSCOR*; Higashi, 1995)	θ_max_ = 27.5°, θ_min_ = 3.1°
*T*_min_ = 0.649, *T*_max_ = 1.000	*h* = −16→16
11855 measured reflections	*k* = −9→9
2970 independent reflections	*l* = −17→17

### Refinement

**Table:**

Refinement on *F*^2^	1 restraint
Least-squares matrix: full	*w* = 1/[σ^2^(*F*_o_^2^) + (0.0125*P*)^2^ + 1.1461*P*] where *P* = (*F*_o_^2^ + 2*F*_c_^2^)/3
*R*[*F*^2^ > 2σ(*F*^2^)] = 0.019	(Δ/σ)_max_ = 0.002
*wR*(*F*^2^) = 0.042	Δρ_max_ = 0.49 e Å^−^^3^
*S* = 1.07	Δρ_min_ = −0.28 e Å^−^^3^
2970 reflections	Absolute structure: Flack (1983), 1417 Friedel pairs
130 parameters	Flack parameter: 0.47 (4)

### Special details

**Table:**

Geometry. All e.s.d.'s (except the e.s.d. in the dihedral angle between two l.s. planes) are estimated using the full covariance matrix. The cell e.s.d.'s are taken into account individually in the estimation of e.s.d.'s in distances, angles and torsion angles; correlations between e.s.d.'s in cell parameters are only used when they are defined by crystal symmetry. An approximate (isotropic) treatment of cell e.s.d.'s is used for estimating e.s.d.'s involving l.s. planes.

### Fractional atomic coordinates and isotropic or equivalent isotropic displacement parameters (Å^2^)

**Table:**

	*x*	*y*	*z*	*U*_iso_*/*U*_eq_	Occ. (<1)
K1	0.38307 (10)	0.50472 (16)	0.30096 (10)	0.0642 (3)	
Nb1	0.023833 (17)	0.05293 (3)	0.03457 (3)	0.01512 (9)	0.861 (4)
V1	0.023833 (17)	0.05293 (3)	0.03457 (3)	0.01512 (9)	0.139 (4)
Nb2	0.313453 (17)	0.07166 (3)	0.03513 (3)	0.01521 (10)	0.889 (4)
V2	0.313453 (17)	0.07166 (3)	0.03513 (3)	0.01521 (10)	0.111 (4)
P1	0.15973 (6)	0.40030 (12)	0.11232 (7)	0.02169 (18)	
S1	0.03047 (5)	0.39473 (11)	0.02326 (8)	0.0258 (2)	
S2	0.05595 (7)	0.15141 (14)	0.40819 (7)	0.0255 (2)	
S3	0.15066 (9)	0.58306 (14)	0.21803 (9)	0.0408 (3)	
S4	0.16690 (6)	0.13978 (11)	0.16577 (6)	0.01913 (18)	
S5	0.29187 (5)	0.41184 (10)	0.02883 (11)	0.03005 (19)	
S6	0.33126 (6)	0.05595 (12)	0.40601 (7)	0.02107 (19)	
S7	0.44830 (7)	0.13335 (13)	0.16897 (6)	0.02327 (19)	
S8	0.60124 (7)	0.10543 (14)	0.39868 (7)	0.0257 (2)	
S9	0.60983 (7)	0.11862 (12)	0.66562 (6)	0.02271 (19)	
S10	0.67360 (6)	0.15938 (11)	0.00015 (6)	0.02081 (17)	

### Atomic displacement parameters (Å^2^)

**Table:**

	*U*^11^	*U*^22^	*U*^33^	*U*^12^	*U*^13^	*U*^23^
K1	0.0831 (8)	0.0470 (6)	0.0625 (8)	0.0035 (6)	−0.0317 (7)	0.0031 (5)
Nb1	0.01049 (12)	0.01887 (15)	0.01601 (14)	−0.00123 (9)	−0.00013 (16)	0.00103 (15)
V1	0.01049 (12)	0.01887 (15)	0.01601 (14)	−0.00123 (9)	−0.00013 (16)	0.00103 (15)
Nb2	0.01068 (13)	0.01820 (15)	0.01675 (14)	0.00116 (9)	0.00020 (16)	−0.00022 (15)
V2	0.01068 (13)	0.01820 (15)	0.01675 (14)	0.00116 (9)	0.00020 (16)	−0.00022 (15)
P1	0.0173 (4)	0.0182 (4)	0.0296 (5)	0.0017 (3)	−0.0002 (3)	−0.0028 (4)
S1	0.0170 (3)	0.0225 (4)	0.0380 (5)	0.0025 (3)	−0.0034 (4)	0.0057 (4)
S2	0.0177 (4)	0.0362 (5)	0.0225 (4)	−0.0038 (4)	−0.0021 (4)	0.0077 (4)
S3	0.0463 (6)	0.0299 (5)	0.0462 (6)	0.0077 (5)	−0.0039 (5)	−0.0184 (5)
S4	0.0164 (4)	0.0221 (4)	0.0188 (4)	0.0005 (3)	0.0004 (3)	−0.0008 (4)
S5	0.0178 (3)	0.0214 (4)	0.0510 (5)	−0.0009 (3)	0.0070 (5)	0.0067 (5)
S6	0.0153 (4)	0.0292 (5)	0.0187 (4)	−0.0010 (3)	0.0004 (3)	0.0031 (3)
S7	0.0203 (4)	0.0279 (5)	0.0216 (4)	0.0006 (4)	−0.0003 (4)	−0.0050 (4)
S8	0.0178 (4)	0.0374 (5)	0.0219 (4)	0.0055 (4)	0.0024 (3)	0.0087 (4)
S9	0.0200 (4)	0.0274 (5)	0.0208 (4)	0.0013 (4)	−0.0012 (3)	−0.0034 (4)
S10	0.0187 (4)	0.0185 (4)	0.0252 (4)	0.0001 (3)	−0.0012 (3)	0.0014 (3)

### Geometric parameters (Å, °)

**Table:**

Nb1—S8^i^	2.4631 (10)	Nb2—S6^i^	2.5490 (9)
Nb1—S7^ii^	2.4760 (9)	Nb2—S10^ii^	2.5551 (8)
Nb1—S2^iii^	2.5039 (10)	Nb2—S5	2.5758 (8)
Nb1—S9^i^	2.5098 (9)	Nb2—S4	2.6279 (8)
Nb1—S6^i^	2.5431 (9)	Nb2—V1^v^	2.8851 (3)
Nb1—S10^ii^	2.5562 (8)	Nb2—Nb1^v^	2.8851 (3)
Nb1—S1	2.5772 (9)	P1—S3	1.9718 (13)
Nb1—S4	2.6319 (8)	P1—S5	2.0451 (13)
Nb1—Nb2^ii^	2.8851 (3)	P1—S1	2.0544 (13)
Nb1—V2^ii^	2.8851 (3)	P1—S4	2.0874 (13)
Nb2—S9^iv^	2.4622 (9)	S2—S8^ii^	2.0235 (15)
Nb2—S2^i^	2.4678 (9)	S6—S10^vi^	2.0498 (12)
Nb2—S8^iv^	2.5110 (10)	S7—S9^iv^	2.0405 (14)
Nb2—S7	2.5406 (9)		
			
S8^i^—Nb1—S7^ii^	111.22 (3)	S9^iv^—Nb2—S7	48.11 (3)
S8^i^—Nb1—S2^iii^	48.07 (4)	S2^i^—Nb2—S7	87.95 (3)
S7^ii^—Nb1—S2^iii^	88.59 (3)	S8^iv^—Nb2—S7	107.57 (3)
S8^i^—Nb1—S9^i^	91.43 (3)	S9^iv^—Nb2—S6^i^	138.35 (3)
S7^ii^—Nb1—S9^i^	48.31 (3)	S2^i^—Nb2—S6^i^	93.10 (3)
S2^iii^—Nb1—S9^i^	107.66 (3)	S8^iv^—Nb2—S6^i^	79.11 (3)
S8^i^—Nb1—S6^i^	89.43 (3)	S7—Nb2—S6^i^	171.57 (3)
S7^ii^—Nb1—S6^i^	141.69 (3)	S9^iv^—Nb2—S10^ii^	91.16 (3)
S2^iii^—Nb1—S6^i^	81.83 (3)	S2^i^—Nb2—S10^ii^	121.84 (3)
S9^i^—Nb1—S6^i^	167.80 (3)	S8^iv^—Nb2—S10^ii^	79.62 (3)
S8^i^—Nb1—S10^ii^	117.95 (3)	S7—Nb2—S10^ii^	137.82 (3)
S7^ii^—Nb1—S10^ii^	94.40 (3)	S6^i^—Nb2—S10^ii^	47.36 (3)
S2^iii^—Nb1—S10^ii^	79.03 (3)	S9^iv^—Nb2—S5	130.13 (4)
S9^i^—Nb1—S10^ii^	140.60 (3)	S2^i^—Nb2—S5	79.09 (3)
S6^i^—Nb1—S10^ii^	47.40 (3)	S8^iv^—Nb2—S5	123.47 (4)
S8^i^—Nb1—S1	79.57 (3)	S7—Nb2—S5	85.19 (3)
S7^ii^—Nb1—S1	128.33 (3)	S6^i^—Nb2—S5	86.79 (3)
S2^iii^—Nb1—S1	125.94 (3)	S10^ii^—Nb2—S5	126.35 (3)
S9^i^—Nb1—S1	82.37 (3)	S9^iv^—Nb2—S4	86.44 (3)
S6^i^—Nb1—S1	85.81 (3)	S2^i^—Nb2—S4	154.57 (3)
S10^ii^—Nb1—S1	125.98 (3)	S8^iv^—Nb2—S4	154.40 (3)
S8^i^—Nb1—S4	155.91 (3)	S7—Nb2—S4	89.85 (3)
S7^ii^—Nb1—S4	86.50 (3)	S6^i^—Nb2—S4	85.62 (3)
S2^iii^—Nb1—S4	152.94 (3)	S10^ii^—Nb2—S4	74.93 (3)
S9^i^—Nb1—S4	88.62 (3)	S5—Nb2—S4	75.48 (3)
S6^i^—Nb1—S4	85.65 (3)	S9^iv^—Nb2—V1^v^	55.30 (2)
S10^ii^—Nb1—S4	74.84 (3)	S2^i^—Nb2—V1^v^	55.11 (2)
S1—Nb1—S4	76.56 (3)	S8^iv^—Nb2—V1^v^	53.78 (2)
S8^i^—Nb1—Nb2^ii^	55.33 (2)	S7—Nb2—V1^v^	53.85 (2)
S7^ii^—Nb1—Nb2^ii^	55.95 (2)	S6^i^—Nb2—V1^v^	132.81 (2)
S2^iii^—Nb1—Nb2^ii^	53.95 (2)	S10^ii^—Nb2—V1^v^	116.74 (2)
S9^i^—Nb1—Nb2^ii^	53.76 (2)	S5—Nb2—V1^v^	115.182 (19)
S6^i^—Nb1—Nb2^ii^	134.72 (2)	S4—Nb2—V1^v^	138.52 (2)
S10^ii^—Nb1—Nb2^ii^	121.07 (2)	S9^iv^—Nb2—Nb1^v^	55.30 (2)
S1—Nb1—Nb2^ii^	110.848 (18)	S2^i^—Nb2—Nb1^v^	55.11 (2)
S4—Nb1—Nb2^ii^	138.22 (2)	S8^iv^—Nb2—Nb1^v^	53.78 (2)
S8^i^—Nb1—V2^ii^	55.33 (2)	S7—Nb2—Nb1^v^	53.85 (2)
S7^ii^—Nb1—V2^ii^	55.95 (2)	S6^i^—Nb2—Nb1^v^	132.81 (2)
S2^iii^—Nb1—V2^ii^	53.95 (2)	S10^ii^—Nb2—Nb1^v^	116.74 (2)
S9^i^—Nb1—V2^ii^	53.76 (2)	S5—Nb2—Nb1^v^	115.182 (19)
S6^i^—Nb1—V2^ii^	134.72 (2)	S4—Nb2—Nb1^v^	138.52 (2)
S10^ii^—Nb1—V2^ii^	121.07 (2)	V1^v^—Nb2—Nb1^v^	0.000 (17)
S1—Nb1—V2^ii^	110.848 (18)	S3—P1—S5	114.14 (6)
S4—Nb1—V2^ii^	138.22 (2)	S3—P1—S1	112.22 (6)
Nb2^ii^—Nb1—V2^ii^	0.000 (18)	S5—P1—S1	111.74 (7)
S9^iv^—Nb2—S2^i^	110.37 (3)	S3—P1—S4	114.43 (6)
S9^iv^—Nb2—S8^iv^	91.42 (3)	S5—P1—S4	100.84 (5)
S2^i^—Nb2—S8^iv^	47.95 (4)	S1—P1—S4	102.37 (5)

Symmetry codes: (i) −*x*+1/2, *y*, *z*−1/2; (ii) *x*−1/2, −*y*, *z*; (iii) −*x*, −*y*, *z*−1/2; (iv) −*x*+1, −*y*, *z*−1/2; (v) *x*+1/2, −*y*, *z*; (vi) −*x*+1, −*y*, *z*+1/2.
